# Is Presence of Bacteria in Preoperative Microscopic Urinalysis of the Patients Scheduled for Cardiac Surgery a Reason for Cancellation of Elective Operation?

**DOI:** 10.5812/aapm.8667

**Published:** 2013-03-26

**Authors:** Mansoor Soltanzadeh, Ahmad Ebadi

**Affiliations:** 1 Department of Anesthesiology, Ahvaz Jundishapur University of Medical Sciences, Ahvaz, Iran

**Keywords:** Cardiac Surgical Procedures, Urinalysis, Pyuria, Bacteria

## Abstract

**Background:**

In some hospitals, urinalysis is done routinely for all patients scheduled for cardiac surgery. Occasionally pyuria or bacteria is reported in the microscopic urinalysis of these patients that are clinically asymptomatic for urinary tract infections.

**Objectives:**

We were seeking answer to this question: is the presence of a different number of bacteria in preoperative microscopic urinalysis of asymptomatic patients scheduled for cardiac surgery indicative of potential postoperative complications and as a result, a good reason to postpone the operation?

**Patients and Methods:**

We conducted a retrospective cross-sectional study based on the review of the medical records of 1165 patients who underwent open-heart surgery.

**Results:**

One hundered and fifty one patients were eligible in our established criteria. There were no significant difference between their demographic characteristics and the same number of randomly selected patients with normal urinalysis who had underwent open-heart surgery. In the bacteriuria group, two patients, and in the control group, three patients had an infection at the operation sites in the post-operative period, which was not a significant finding between two groups (P = 0.503).

**Conclusions:**

We recommend that in the absence of symptoms of urinary tract infection, urinalysis is not necessary and not cost beneficial in the preoperative evaluation of patients scheduled for open-heart surgery.

## 1. Background

Open-heart surgery is one of the major operations in which the use of cardio-pulmonary bypass circulation by extracorporeal devices causes wide-spread of pathophysiological changes in the vital organs of the human body during and following the operation. Because of these vast changes in the mechanical, physiological and biochemical functions of the patient's body in perioperative period, the operation team especially anesthesiologists and cardiac surgeons need comprehensive preoperative evaluations of patient's status. In some hospitals such as our hospital, urinalysis is one of the laboratory tests that is done routinely prior to cardiac surgery routinly. The urine culture does not include routine laboratory tests in the absence of any symptom of urinary tract infection in the preoperative assessment of the candidates for open-heart surgery and it is only ordered when symptoms of urinary tract infection are presented. Occasionally pyuria or bacteria is reported in the microscopic urinalysis of patients who are clinically asymptomatic for urinary tract infection. There are some references regarding preoperative management of asymptomatic bacteriuria in different kinds of surgeries such as joint operations (1, 2). However we did not find any comment in the litrature for open-heart surgery regarding preoperative management of patients with a significant bacterial count (usually ≥ 100000 colony forming units [CFU] /mL) in the urine culture or the presence of bacteria in their preoperative microscopic urinalysis in the absence of symptoms of urinary tract infection.

The urinary tract is normally sterile except for the flora in the last few centimeters of the distal urethra, which is varied and reflects the existence of digestive flora (enterobacteria, streptococci, anaerobic bacteria), the skin flora (coagulase negative staphylococci, corynebacteria), and the genital flora (lactobacilli in female patients) (3). Asymptomatic bacteriuria is defined as a significant bacterial count (usually ≥ 100000 colony forming units [CFU] /mL) present in the urine of subjects without any urinary symptoms (4, 5). However, in some litrature, five bacteria per high power field (HPF) represent roughly 100,000 colony-forming units (CFU) per mL (6). In major operations like open-heart surgery, preoperative UTI can cause some post-operative complications such as exacerbation of UTI (7), septicemia (8), endocarditis and infections alongside the operations (9, 10). It is notable that factors such as attenuation of immune system following extracorporeal circulation, diabetes mellitus and indwelling urinary catheter in this kind of operation may increase the risk of such complications.

## 2. Objectives

We were seeking an answer to this question: is the presence of a different numbers of bacteria in preoperative microscopic urinalysis of asymptomatic patients scheduled for cardiac surgery an indicator for the likelihood of postoperative complications and a reason for postponing the operation?

## 3. Patients and Methods

We conducted a retrospective cross-sectional study based on medical records of Ahvaz Imam Khomeini hospital. We reviewed the medical charts of 1165 patients who underwent open-heart surgery between March 2010 and March 2012. We enrolled the medical charts of patients that were asymptomatic for urinary tract infections, but had bacteriuria with or without pyuria in microscopic urinalysis and compared them with the same number of randomly selected patients with normal urinalysis who had underwent open-heart surgery in our hospital during the same period as the control group. In microscopic urinalysis, microorganisms are usually reported as "none", "few", "moderate" and "many" present per high power field (HPF). We included those medical charts that had "moderate" or "many" bacteria in urinalysis reports. Men normally have fewer than two white blood cells (WBCs) per HPF; and women normally have fewer than five WBCs per HPF in urinalysis (5); therefore we included the persons whom urinalysis was more than 4 WBC/HPF for men and more than 6 WBC/HPF for women. We reviewed the medical charts of the patients for postoperative septicemia, endocarditis, symptomatic urinary tract infections (UTI) and infection of the operation sites during the time of postoperative ICU admission until discharge from the hospital. Patients were followed until two months following discharge with a review of patient's records if hospitalized again for one of the above complications. As the catheter is the source of infection, the best way to treat nosocomial urinary tract infections is by removing the catheter. By such a simple method, up to two thirds of bacteriuria in patients with a catheter will be cleared within a week (11). As result, we excluded medical records of the patients who had urinary catheter more than five days. We also excluded subjects with traumatic urethral catheterization. Perioperative antibiotic therapy regardless of bacteria in urinalysis were done for all patients underwent open-heart surgery which is a routine protocol ordered by cardiac surgeons. Statistical analyses were done with SPSS version 17.0 with presenting numerical data as means and standard deviations. Comparison between the groups was performed using the unpaired student's t-test and the chi-squared and variance analysis. A P value of less than 0.05 was considered statistically significant.

## 4. Results

In the review of patients' medical records, 151 patients (of 1165 patients) were eligible according our established criteria and compared with the same number of randomly selected patients with normal urinalysis who had underwent open-heart surgery in our hospital during the same time duration. The demographic characteristics between two groups are presented in Table 1, which revealed no significant differences between two groups. In the bacteriuria group, two patients had an infection, one at the operative skin incision of sternum and the other one at skin incision of the leg in the post-operative period. In the control group, three patients had an infection, two at the operative skin incision of the sternum and one at the skin incision of leg in the post-operative period, but there were not any significant differences between two groups (P=0.503). In the review of medical records of both groups, there were no reports of infections at the other sites of the body like urinary tract and heart valves. On the basis of gender distribution, 86 male and 65 female patients had bacteria in their urinalysis (Table 2). From 151 patients with bacteriuria, 97 cases (64.24%) had bacteriuria with pyuria and 54 patients (35.76%) had bacteriuria without pyuria. From 151 patients with bacteriuria, 102 cases (67.5%) had moderate and 49 patients (32.5%) had many microorganisms in microscopic urinalysis.

**Table 1:** Comparison of Demographic Characteristics of Patients Considered between Two Groups

Variable	Bacteriuria Group (n = 151)	Control Group (n = 151)	P value
**Age, y, Mean **±** SD**	68.4 ± 12.4	66.2 ± 11.2	0.150
**Weight, kg, Mean **±** SD**	62.6 ± 9.8	64.1 ± 11.3	0.215
**Gender, Male/Female, No.**	86/65	79/72	0.155

**Table 2:** Patients Distribution on Basis of Gender

Gender	Whole patients on the basis of gender, No. (%)	Patients with bacteriuria, No. (%)	Male or Female with Bacteriuria /Whole Patients of the same gender, %
**Male**	781 (67.03)	86 (56.96)	11.01
**Female**	384 ( 32.96)	65 (43.04)	16.92

## 5. Discussion

Bacteria are common microorganisms in urine specimens because of their abundant normal microbial flora of the vagina or external urethral meatus and because of their ability to rapidly multiply in urine standing at room temperature. Therefore, microbial organisms found in all but the most scrupulously collected urines should be interpretive in view of clinical symptoms. The most accurate test for bacteriuria is urine culture. The most commonly used tests for detecting bacteriuria in asymptomatic persons are dipstick urinalysis and direct microscopy (12). Microscopic results have been found to be of questionable value for screening. Bailey (12) determined that microscopic detection of moderate numbers of bacteria and leukocytes in the urine had sensitivities of less than 75% and 85%, respectively. The specificity for a combination of both tests was less than 85%. However the positive predictive value of microscopic examinations for pyuria, bacteriuria, or both has been shown to be as low as 33% (13). It seems that while urinalysis and urine microscopic examination are often used to collect evidence for or against a urinary tract infection, none of the components of these tests should be relied to reach diagnosis. Lin et al. (14) reported a sensitivity of 64.9% for standard urinalysis. Similarly, several studies indicate that the sensitivity of standard urinalysis is low (15, 16). In our study, two patients of asymptomatic bacteriuria group and three patients of non bacteriuria group (control group) had infection, at the operative skin incision site in the post-operative period, which was not a significant difference between two groups. Therefore it does not seem that there was a link between bacteriuria and skin infections. Notably, of two patients with skin infection in asymptomatic bacteriuria group, one had moderate and another had numerous microorganisms in microscopic urinalysis. Another important point is that all of five patients with skin infections two in bacteriuria group and three in non-bacteriuria group had diabetes mellitus in their past medical history. Following an indwelling urinary catheter, the patients’ colonic flora rapidly colonizes the urinary tract in these patients (17). Stark and Maki (18) have demonstrated that in catheterized patients, bacteria in the urinary system quickly proliferate to exceed 105 CFU/mL over a short period of time. Bacteriuria, defined as a quantitative culture of ≥ 105 CFU/mL, has been reported in up to 30% of catheterized hospitalized patients (19). The terms “bacteriuria” and “UTI” are generally, although incorrectly, used as synonyms. Furthermore, the presence of white cells in the urine is not useful for differentiating colonization from infection, as most catheter-associated bacteriurias have pyuria alongside themselves (20). In our study, there were no clinical symptoms of urinary tract infections regardless of indwelling urinary catheter in post-operations in asymptomatic patients with abnormal urinalysis. In our study from 151 bacteriuria cases, 86 male and 65 female had bacteria in their urinalysis. The prevalence given from asymptomatic bacteriuria is higher in the women than the men. Athough the reason for more bacteriuria in men than the women in our study is that only 384 from 1165 patients (32.96%) were women. According to our findings, the urinalysis cannot be relied on as a sole test in the absence of symptoms of urinary tract infection (fever > 38 °C, urinary urgency, pollakiuria, burning sensation or suprapubic pain on voiding) and is not a sufficiently strong predictor of urinary tract infection. The negative predictive value is too low to permit the physician to confidently rule out urinary tract infection, while the positive predictive value is too low to confirm diagnosis of a urinary tract infection therefore it was deemed unnecessary to postpone the operation. In the certain hospitals, prophylactic intravenous antibiotic therapy is started with an antibiotic like ceftriaxone (2 gr iv.) prior open heart surgery in the preoperative management of the patients with a significant number of bacteria in their urinalysis tests in the absent of clinically any symptoms of urinary tract infection. However we recommend that in the absent of urinary tract infection symptoms in preoperative evaluation of patients scheduled for open-heart surgery, urinalysis is not necessary and not cost benefitial.
